# Assessing the efficacy of perilymphatic fistula repair surgery in alleviating vestibular symptoms and associated auditory impairments

**DOI:** 10.3389/fneur.2023.1269298

**Published:** 2023-10-12

**Authors:** Han Matsuda, Jeremy Hornibrook, Tetsuo Ikezono

**Affiliations:** ^1^Department of Otorhinolaryngology, Faculty of Medicine, Saitama Medical University, Saitama, Japan; ^2^Department of Otolaryngology-Head and Neck Surgery and Audiology, Christchurch Hospital, Christchurch, New Zealand

**Keywords:** dizziness, vertigo, disequilibrium, postural instability, unsteadiness, PLF repair surgery, CTP, Cochlin-tomoprotein

## Abstract

Perilymph Fistula (PLF), abnormal communication between the fluid-filled space of the inner ear and the air-filled space of the middle ear, is a significant cause of vestibular and auditory symptoms. This is a retrospective study of 22 cases treated with PLF repair surgery, selected based on our surgical indication. We analyzed the characteristics of these 22 cases and evaluated the efficacy of PLF repair surgery in treating vestibular and auditory symptoms. Cases with antecedent events had significantly shorter intervals before surgery. The postoperative recovery from vestibular symptoms following PLF repair surgery was strikingly rapid, with 82% of cases demonstrating marked improvement within a week, even in chronic cases. Despite the notable absence of a control group in the study, the marked improvements in vestibular symptoms and substantial reductions in Dizziness Handicap Inventory (DHI) scores suggest that the observed benefits are attributable to the surgical intervention. Further, timely surgery showed improvements in hearing, with some benefits also seen in late-stage surgeries. Using the perilymph-specific protein Cochlin-tomoprotein (CTP) as a diagnostic biomarker, we could prove that PLF could be responsible for disequilibrium and related auditory disturbances in these patients. A new hypothesis is proposed that the chronic disequilibrium experienced by many PLF patients is due to enhanced mobility of the utricle and not to endolymphatic hydrops. Further research is needed to fully elucidate PLF’s symptoms and treatment efficacy using the surgical indication we developed.

## Introduction

Perilymph fistula (PLF) is an abnormal communication between the fluid (perilymph)-filled inner ear and the air-filled space of the middle ear and mastoid, or cranial spaces. PLF is a well-known cause of vestibular and auditory symptoms, and there are many reports on treatment outcomes, and a high cure rate has been reported, especially for the vestibular symptoms (1, 2). We have noted that patients presenting with suspected PLF-induced disequilibrium, even those with longstanding intractable chronic disequilibrium, often demonstrate significant and rapid improvements following PLF repair surgery. This observation has been recurrently emphasized in academic discussions among specialists.

Despite those previous experiences, this fact has yet to be fully documented in scientific literature. There needs to be a standardized, established treatment for vestibular symptoms of PLF. One reason for this unfortunate situation is that there was no definitive diagnostic method to identify the perilymph leakage (3, 4). We have identified an isoform of Cochlin, Cochlin-tomoprotein (CTP), as a perilymph-specific protein that is not expressed in the blood, CSF, or saliva (5). We have reported the specificity and sensitivity of the CTP test (6) and the clinical characteristics, epidemiology, and anatomic leakage routes of PLF diagnosed with the CTP test (7, 8). The Japanese FDA approved this test, which became eligible for national insurance coverage in Japan in 2022. The advent of the CTP test has enabled a scientific appraisal of this condition, long dubbed as controversial.

The principal objectives of this investigation are threefold. First, we aim to understand the features of cases selected based on our surgical indication. Second, our focus shifts to evaluating the outcomes of cases post-PLF repair surgery. This includes examining the duration until noticeable improvement in disequilibrium issues is observed and significant changes in the Dizziness Handicap Inventory (DHI) before and after the procedure. Within this context, we are also keen on assessing the degree of auditory enhancement and identifying specific characteristics of cases that show notable progress. Lastly, we intend to introduce and gauge the efficacy of our recently developed, highly specific CTP detection test designed to pinpoint perilymph leakage.

## Materials and methods

This study involves a retrospective review of patient records of individuals who underwent PLF repair surgery between July 2011 and May 2019 at our tertiary referral hospital. All the cases were checked by CT scan by 2 certified radiologists, and none of them showed any evidence of otic capsule dehiscence. Of the 37 cases that underwent surgery during this timeframe, we excluded 4 cases involving direct stapes injury and 11 presenting solely cochlear symptoms. The study concentrates on analyzing the remaining 22 cases exhibiting vestibular symptoms. To evaluate subjective vestibular symptoms, we conducted interviews with the patients. These interviews aimed to ascertain whether patients experienced rotational vertigo or other symptoms, such as disequilibrium, postural instability, and unsteadiness. DHI was also utilized. Furthermore, the study examined the duration of symptoms from onset until surgery, evaluated the presence of nystagmus through positional and positioning nystagmus tests using an infrared camera, determined auditory thresholds using the pure tone average (PTA), the arithmetic mean of five frequencies (0.25, 0.5, 1, 2, and 4 kHz) from audiometric testing and utilized a CTP detection test (explained below). vHIT (head impulse test) and VEMP (Vestibular Evoked Myogenic Potential) were performed on some patients but not all; hence, we excluded those results from the whole group analysis.

We categorized the cases into four groups based on antecedent events and underlying causes, following the criteria formulated by a Japanese study group (Table 1) (7). Category 1 is limited to cases linked with a history of head trauma from traffic accidents. While the original category encompassed direct trauma to the inner/middle ear and middle/inner ear diseases and surgeries, these cases are beyond the purview of this study and are therefore excluded. Category 2 comprised cases with barotraumas stemming from external factors, including activities such as flying and diving. Category 3 encompassed cases with barotraumas linked to internal factors, such as nose-blowing and straining. Category 4 was designated for idiopathic cases, which lacked any discernible antecedent events. A comprehensive interview was conducted to chronicle the patient’s medical history, with particular attention to events preceding the onset of symptoms. Pre-operative care included conservative treatment employing standard methodologies. A combination of corticosteroids, isosorbide, vitamin B12, and adenosine triphosphate was administered for cases of hearing loss. We instruct patients to avoid the following activities: external barotraumatic events, which typically include hyperbaric oxygen therapy, flying, diving, and rapid altitude changes; and internal barotraumatic events, which include lifting heavy objects, blowing their nose strongly, straining during constipation (for which they should take prescribed pills), solid coughing, engaging in sexual activity, and performing intense physical exercise. We recommend that they remain in bed at a 30° elevated position for 1 week if possible. For cases exhibiting vestibular symptoms, betahistine and diphenidol were prescribed. All patients with a history of symptoms lasting longer than 3 months were advised to undertake a home-based vestibular rehabilitation program (9).

**Table 1:** Categorization of PLF cases formulated by a Japanese study group.

Category 1
Linked to trauma, middle, and/or inner ear diseases, surgeries
1) a Direct labyrinthine trauma (stapes luxation, otic capsule fracture, etc.)
b Other trauma (head injury, body contusion, etc.)
2) a Middle or inner ear diseases (cholesteatoma, tumor, anomaly, dehiscence, etc.)
b Iatrogenic (ear surgeries, medical treatments, etc.)
Category 2
Linked to barotrauma caused by antecedent events of external origin
(such as flying or diving)
Category 3
Linked to barotrauma caused by antecedent events of internal origin
(such as straining, sneezing, or coughing)
Category 4
Has no apparent antecedent event (idiopathic)

“Spontaneous” should not be used.

Our indication for PLF repair surgery to address vestibular symptoms were as follows (Table 2A): (1) the patient having a history of traumatic or barotraumatic events before the onset of vestibular symptoms, and (2) In idiopathic patients whose chief complaint is sustained disequilibrium, the onset or exacerbation of vestibular symptoms were accompanied by sudden, fluctuating (both improvement and deterioration of more than 10 dB in PTA), and progressive (deterioration of more than 10 dB) hearing loss. The sustained vestibular symptoms are disequilibrium, unsteadiness, and postural instability, swaying, all henceforth referred to as disequilibrium. The surgery was conducted under general anesthesia, utilizing a transcanal approach with the elevation of the tympanomeatal flap, facilitating the visualization of the middle ear. Connective tissue and fibrin glue were used to seal the round and oval windows, microfissures around windows (10), and the fissula ante fenestram. Thin aural cartilage was placed over the round window to stabilize the connective tissue. Throughout the surgical procedure, meticulous examination was conducted to ascertain the presence or absence of a fistula and any perilymph leakage.

**Table 2:** Surgical indication **(A)** and the number of cases in each characteristic **(B)**.

(A)
Group1: cases in Category 1, 2, and 3
Having a history of traumatic or barotraumatic events before the onset of vestibular symptoms.
Group 2: cases in category 4 (idiopathic)
Having sustained vestibular symptoms (disequilibrium, unsteadiness, postural instability, and swaying)
The onset or exacerbation of vestibular symptoms was accompanied by sudden, fluctuating, and progressive hearing loss.

(B)		
Group 1: cases in category 1, 2, and 3		
1A	Sudden hearing loss	4 cases
1B	Progressive/fluctuating hearing loss	9 cases
1C	without hearing loss	2 cases
Group 2: cases in category 4 (idiopathic)		
2A	Sudden hearing loss	4 cases
2B	Progressive/fluctuating hearing loss	3 cases

Group 1, which encompasses Categories 1, 2, and 3, and Group 2, which includes Category 4 cases. Subgroup A encompassed cases exhibiting sudden hearing loss, Subgroup B included cases with progressive or fluctuating hearing loss, whereas Subgroup C consisted of cases without hearing loss.

After the PLF repair surgery, improvement in vestibular symptoms was assessed through patient interviews, and the results were binarily categorized as either unchanged/progressing or demonstrating marked improvement compared to the pre-surgery state. Evaluations of vestibular symptoms and nystagmus were systematically conducted at 1 week, 1 month, 3 months, and 6 months after the surgery. The moment when vestibular symptoms noticeably decrease and patients report symptom relief is identified as the point of marked improvement. The time frame in which patients experienced marked symptom improvement and resolution of nystagmus was classified as follows: A—within 1 week postoperatively, B—within 1 month postoperatively, C—within 3 months postoperatively, D–within 6 months postoperatively, E—no observable improvement in vestibular symptoms or resolution of nystagmus. DHI score was evaluated prior to surgery and either at the resolution of vestibular symptoms or at a six-month post-operative period, whichever came first. Of the 22 cases, DHI assessment was executed in 12 cases that underwent surgery post-September 2015. Although the video head impulse test and vestibular evoked myogenic potentials were performed, these tests were not conducted in all cases; therefore, the data gathered from these tests were not incorporated into the analysis. PTA was evaluated once auditory stability was achieved, defined by a constant PTA that did not exhibit variations beyond a 10 dB threshold. The evaluation of auditory recovery followed the criteria established by the Acute Severe Hearing Loss Study Group, which are as follows: “Complete recovery” signifies recovery to a hearing level within 20 dB at PTA or to the same hearing level as the unaffected side. “Marked recovery” refers to more than 30 dB recovery in PTA. “Slight recovery” is a recovery of 10–29 dB, and “No response” corresponds to a recovery of less than 10 dB (11). A binary statistical analysis was executed to classify treatment responses as either positive (improvement exceeding 10 dB) or negative (improvement of less than 10 dB or a decline) in PTA.

The CTP detection test was conducted as previously detailed (7). Briefly, following myringotomy or during surgery, the middle ear was irrigated with 0.3 mL of saline and the fluid retrieved was termed Middle Ear Lavage (MEL). The MEL was frozen, sent to the central pathology lab (SRL Inc.), then subjected to analysis using the ELISA method with polyclonal antibodies (6). CTP concentrations equal to or greater than 0.8 ng/mL were deemed positive; values between 0.4 and 0.8 ng/mL were considered intermediate, and levels below 0.4 ng/mL were classified as negative. A positive CTP level led to the definitive diagnosis of PLF.

Patient data was thoroughly analyzed, and follow-ups were conducted until the resolution of vestibular symptoms or at a six-month post-operative period, whichever came first. Statistical analysis was performed using JMP Pro 16 software. The Wilcoxon test was employed to compare pre-and post-operative DHI scores. At the same time, Spearman’s rank correlation coefficient was utilized to analyze the relationship between hearing improvement and the number of days. A value of p less than 0.05 was taken as indicative of statistical significance. The Institutional Review Board (IRB) of Saitama Medical University Hospital approved this study (approval number 13-086).

## Results

Based on the established categorization criteria (as shown in Table 1) and a comprehensive interview and exclusion process, 22 patients were included in this study, diagnosed as suspected cases of PLF, and considered for surgical intervention. As detailed in the Materials and Methods section and Table 2A, our surgical indication is based on the sustained vestibular symptom, antecedent traumatic events, and/or associated hearing loss (sudden, progressive, or fluctuating). Table 2B summarizes the number of cases in each characteristic. Given the limited number of patients, we conducted a bifurcated analysis by Group 1 (Category 1, 2, 3; Table 3A) and Group 2 (Category 4; Table 3B). The demographics were summarized in Table 4, and detailed patient numbers in groups 1 and 2 are summarized in Supplementary Table.

**Table 3:** Clinical features of cases in Group 1 **(A)** and Group 2 **(B)**.

(A)																	
No.	Age	Gender	Category	Event	Days from onset to surgery	CTP test	Vestibular symptom	Improvement in vestibular symptom	Resolution of nystagmus	Hearing type	Preop DHI	Postop DHI	Preop PTA	Postop PTA	Hearing recovery	Follow-up period	Surgical indication
1	49	F	3	Nose blowing	5	I	D	A	C	-	16	0	NOR	NOR	-	13	1C
2	64	F	2	Airplane	8	N	R-D	A	A	S	NA	NA	111	111	NR	6	1A
3	46	M	3	Blow the whistle	11	N	D	A	B	P	62	0	104	67	MR	3	1B
4	33	M	2	Airplane	12	N	R-D	C	A	P	NA	NA	89	49	MR	11	1B
5	13	M	3	Nose blowing	14	N	D	A	A	P	20	0	101	72	SR	23	1B
6	32	F	3	Nose blowing	18	P	R-D	A	A	F	96	0	74	58	SR	9	1B
7	65	F	3	Airplane	27	N	D	A	A	P	76	2	110	87	SR	14	1B
8	52	M	2	Descent from high altitude	35	P	R-D	A	B	F	58	10	101	86	SR	17	1B
9	32	F	3	Sexual intercourse	69	I	D	A	-	P	NA	NA	98	92	NR	53	1B
10	47	F	1	Traffic accident	102	N	D	A	C	S	NA	NA	37	22	SR	15	1A
11	39	F	2	Airplane	136	P	D	A	-	-	NA	NA	NOR	NOR	-	2	1C
12	66	M	2	Insufflation	150	I	D	A	A	F	NA	NA	31	35	NR	36	1B
13	67	M	3	Vomit	167	N	D	E	E	S	78	48	68	78	NR	12	1A
14	45	F	3	Nose blowing	179	N	R-D	A	A	S	NA	NA	58	60	NR	3	1A
15	62	M	1	Traffic accident	190	I	D	A	-	P	NA	NA	53	36	SR	1	1B

(B)															
No.	Age	Gender	Days from onset to surgery	CTP test	Vestibular symptom	Improvement in vestibular symptom	Resolution of nystagmus	Preop DHI	Postop DHI	Hearing type	Preop HL	Postop HL	Hearing recovery	Follow-up period	Surgical indication
16	39	M	41	N	R-D	B	D	NA	NA	S	111	70	MR	46	2B
17	44	F	77	N	D	A	A	62	0	P	111	111	NR	30	2B
18	48	M	122	N	R-D	A	D	44	0	P	56	48	NR	6	2B
19	74	F	215	N	D	E	E	54	42	S	82	89	NR	11	2A
20	47	F	290	N	D	A	-	80	0	S	60	64	NR	14	2A
21	55	M	390	N	D	A	-	NA	NA	S	59	62	NR	1	2A
22	47	M	1,500	P	R-D	A	-	92	68	S	98	104	NR	9	2A

The cases were numbered in order of the duration from onset to surgery. The patients were categorized into four groups based on antecedent events and underlying causes (Category, Event). The Days represent the number of days from onset to surgery. CTP examination results were indicated as positive (P), intermediate (I), or negative (N). Vestibular symptoms were classified as follows: cases initially complained of rotatory vertigo and turned into disequilibrium (R-D), and cases originally presented as disequilibrium (D). The time frame in which patients experienced marked symptom improvement and resolution of nystagmus was classified as follows: A—within 1 week postoperatively, B—within 1 month postoperatively, C—within 3 months postoperatively, D—within 6 months postoperatively, E—no observable improvement in vestibular symptoms or nystagmus persisted. A minus sign (−) was recorded in the nystagmus column for cases without preoperative nystagmus. The hearing type was classified as sudden (S), progressive (P), or fluctuating (F). Preoperative and postoperative PTA were measured as the average air conduction thresholds at 0.25 to 4 kHz frequencies. NOR indicates normal hearing. Hearing recovery was classified as marked recovery (MR), slight recovery (SR), and no response (NR). The follow-up period represents the duration of postoperative follow-up (months). Surgical indications were documented based on (Table 2B). Group 1; Among the 15 cases, marked improvement of vestibular symptoms within 1 week was observed in 13 cases. Group 2; Among the 7 cases, marked improvement of vestibular symptoms was observed within 1 week in 5 cases. A statistically significant shorter interval before surgery was observed in Group 1 than in Group 2.

**Table 4:** Demographics of the enrolled patients.

	Overall (22 cases)	Group 1 (Table 3A)	Group 2 (Table 3B)
Gender	11 males, 11 females	7 males, 8 females	4 males, 3 females
Age range	13 to 74 years	13 to 67 years	39 to 74 years
Median age	47 years	47 years	47 years
Duration from Symptom onset to surgery^*^	5 days to ~4 years, Median: 89.5 days	5 to 190 days, Median: 35 days^*^	41 days to ~4 years, Median: 215 days

^*^Statistical significance. Shorter interval before surgery (*p* < 0.05, Wilcoxon test).

Among the studied population, preceding incidents were head injury due to a traffic accident (Category 1), airplane flying, descent from a high-altitude mountain, insufflation (Category 2), nose-blowing, blowing the whistle, sexual intercourse, and vomiting (Category 3). Table 3 systematically organizes the patients in descending order according to the period from the onset of symptoms to the surgical intervention. The duration varied from 5 days to approximately 4 years, with a median of 89.5 days. A statistically significant shorter interval before surgery was observed in Group 1 (*p* < 0.05, Wilcoxon test). Five patients underwent surgery during the acute phase (within 2 weeks post-symptom onset). Ten patients experienced a chronic phase of vestibular symptoms lasting 90 days or longer.

**Figure 1:**
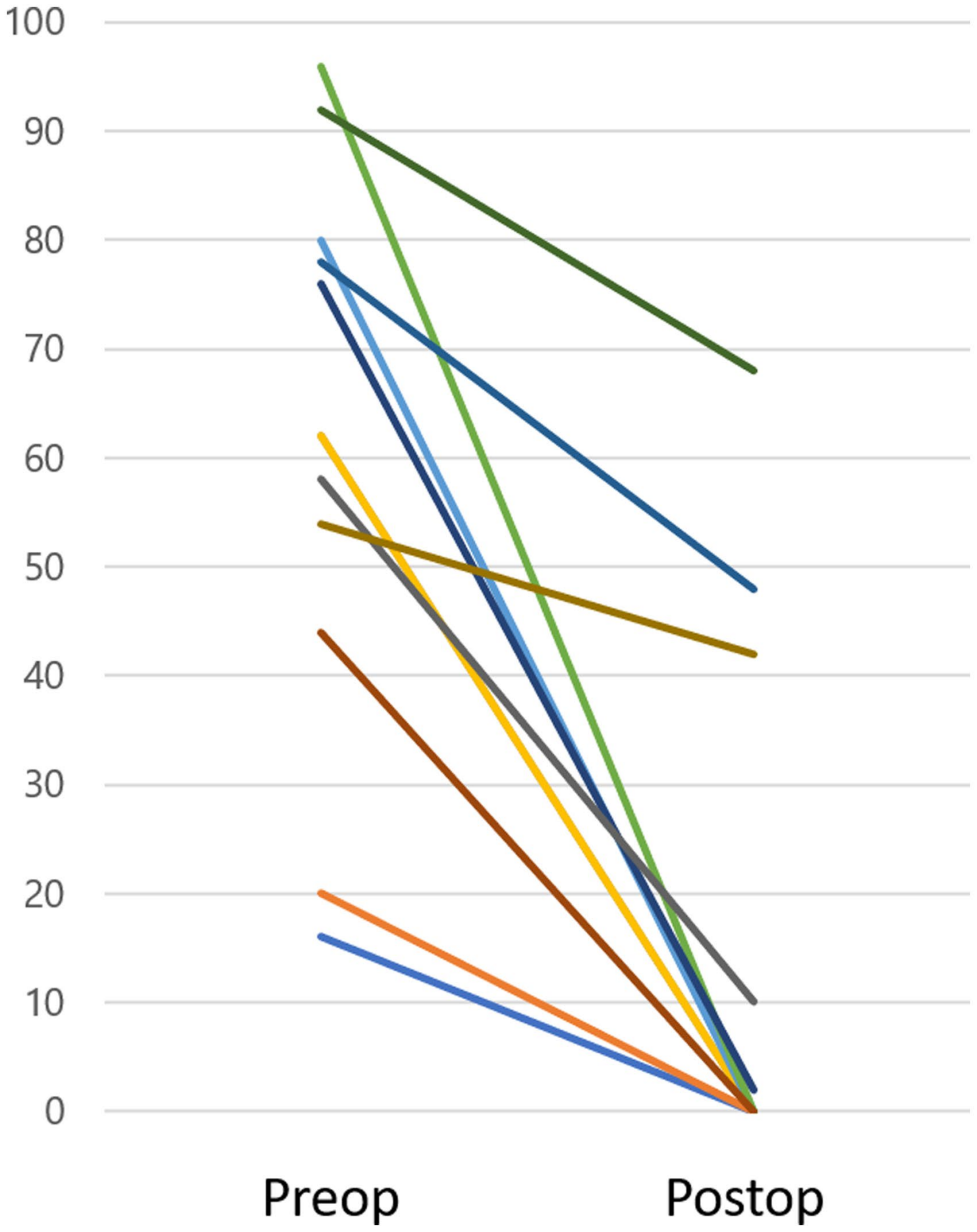
DHI score. Preoperative DHI scores ranged from 16 to 96, while postoperative scores were between 0 and 68, demonstrating a significant improvement after surgery (*p* < 0.05). Three cases (Case 13, 19, and 22) sustained a DHI score over 10 (48, 42, 68, respectively) within the observation period.

CTP test outcomes varied across the patient population: 4 patients had positive results, 4 had intermediate results, and 14 demonstrated negative results. Only one instance (case 6) (8) showed a visible intraoperative fistula due to a malformed stapes footplate and minimal perilymph leakage. In contrast, in the remaining 21 cases, neither fistula nor microfissure was detected, nor was any noticeable perilymph leakage observed intraoperatively. Eight patients initially experienced rotatory vertigo, subsequently evolving into disequilibrium within a 1 to 7-day timeframe. In contrast, 14 patients did not experience initial rotatory vertigo, presenting with disequilibrium at onset. MRI findings in the affected ear revealed an inner ear hemorrhage in case 13 and fibrosis in the posterior semicircular canal in case 19. These 2 cases were intractable in several measures, subjective vestibular symptom, DHI, nystagmus and hearing, as explained below. Within a week post-surgery, 18 out of the 22 patients (82%) demonstrated marked improvements in vestibular symptoms (Table 5). Among the remaining 4 patients, one experienced marked improvement a month after surgery, while another observed this change 3 months post-surgery. Two patients (cases 13 and 19) failed to achieve marked improvement 6 months after the surgical intervention. Peripheral nystagmus was observed in 16 of the 22 patients before surgery. All cases displayed unidirectional rotatory-horizontal nystagmus during the positioning nystagmus test. Additionally, Case 13 exhibited down-beating rotatory nystagmus in the Dix-Hallpike test at the onset. Postoperatively, nystagmus subsided in 8 patients after 1 week, 2 patients after 1 month, 2 patients after 3 months, and 2 patients after 6 months. However, 2 patients (cases 13, 19) continued to exhibit nystagmus 6 months after surgery. DHI scores varied between 16 and 96 preoperatively and 0–68 postoperatively, showing a statistically significant improvement post-surgery (*p* < 0.05, Wilcoxon test), as depicted in Figure 1.

**Table 5:** The interval from surgery to the point of marked improvement in vestibular symptoms.

Point of resolution	The number of cases
Within 1 W	18
1 M	1
3 M	1
No change	2

Within a week post-surgery, 18 out of the 22 patients (82%) demonstrated marked improvements in vestibular symptoms. Among the remaining four patients, one experienced marked improvement a month after surgery, while another observed this change 3 months post-surgery. Notably, two patients (13 and 19) failed to achieve marked improvement 6 months after the surgical intervention.

Excluding two cases with normal hearing (cases 1 and 11), hearing loss was identified in 20 patients. Nine patients experienced sudden onset, 3 had fluctuating hearing loss, and 8 had progressive hearing loss. Postoperative hearing improvements ranged from a decrease of 10 dB to an increase of 41 dB, with a median improvement of 7 dB. The only case where hearing deteriorated by more than 10 dB was Case 13, which had inner ear hemorrhage. There were no instances of complete recovery. However, marked recovery was observed in 3 cases, slight recovery in 6 cases, and no recovery was found in 11 cases. Patients who demonstrated a postoperative response to the treatment, reflected by a hearing improvement of 10 dB or more (either marked or slight recovery), had a statistically significantly shorter interval from symptom onset to surgery (median 27 days) compared to those who exhibited no response (median 167 days; *p* < 0.05 Spearman’s rank correlation coefficient).

## Discussion

Drawing from our experiences, we have established indication for PLF repair surgery to address vestibular symptoms. The indication are: (1) the patient has a history of traumatic or barotraumatic events preceding the onset of vestibular symptoms, and (2) vestibular symptoms are accompanied by sudden, fluctuating, or progressive hearing loss, as explained in the method section (Table 2). In Group 1, the patients exhibited specific antecedent events that are characteristic indicators for PLF. Given these clinical histories, the likelihood of diagnosing PLF becomes comparatively more straightforward. Our study found a statistically significant shorter interval before surgery in Group 1 patients, compared to Group 2 patients without a history of antecedent events. This is reasonable because Group 2 patients were referred to our clinic for sustained disequilibrium. In Group 1, 20% of the cases (3 out of 15) tested positive for CTP, aligning with the positive rate discovered in our prior nationwide study. That study reported positive rates of 32% for Category 1, 11% for Category 2, and 24% for Category 3 cases, all of which led to a confirmed diagnosis of PLF. Most cases (13 out of 15) had an abrupt onset of vestibular and auditory symptoms following these events, excluding two cases with normal hearing (cases 1 and 11). PLF repair surgery was chosen due to the apparent relationship between the event and onset in case 1, the preoperative positive CTP test result in case 11. In Group 2, comprising idiopathic cases, only one case (1/7, 14%) tested positive for CTP. Although this category of PLF is a matter of ongoing debate, our previous nationwide study confirmed its existence, with 18% of CTP-positive cases being verified as PLF in Category 4, idiopathic. The hearing progression in patients with PLF can present with various types of hearing impairment, including sudden or progressive/fluctuating sensorineural hearing loss. The presence of these auditory symptoms, together with vestibular disturbances, could serve as an indicator for clinicians to suspect PLF (7). Hence, diagnosing PLF without cochlear signs or a traumatic history can be challenging. Our study did not include such isolated cases.

Determining how to include PLF-suspected cases based on specific vestibular/cochlear symptoms is paramount. To date, the classic symptoms and signs that conclusively link to a PLF diagnosis remain unidentified. In our research, 8 patients initially exhibited rotatory vertigo, which later transitioned into disequilibrium. Meanwhile, the other 14 cases did not have initial rotatory vertigo but presented with disequilibrium from the onset. Hearing loss patterns varied, ranging from sudden onset to fluctuating and progressive patterns. Thus, during patient interviews, the pivotal question is: “Do you recall any preceding traumatic or barotraumatic events that could correlate with the commencement of your current vestibular/cochlear symptoms?” For idiopathic patients whose chief complaint is sustained disequilibrium, the central focus is determining if the onset or exacerbation of vestibular symptoms is aligned with acute hearing loss or with progressive/fluctuating hearing loss. In our analysis, 5 out of the 7 idiopathic cases experienced acute hearing loss just before symptom onset, while the remaining 2 reported progressive or fluctuating hearing loss.

A prior animal study provides potential explanations for those variable vestibular symptoms, highlighting diverse pathologies in the membranous labyrinth (12). For example, experimental fistulae in guinea pigs led to the collapse of the semicircular canal ampullae, potentially causing rotatory vertigo, and hydrops or collapse of the otolithic organs. As such, PLF patients may exhibit a spectrum of symptoms due to these varied inner ear conditions.

The postoperative recovery from vestibular symptoms following PLF repair surgery was strikingly rapid, with 82% of cases demonstrating marked improvement within a week (87% in Group 1, 71%in Group 2). All eight patients with positive or intermediate CTP and 10 out of 14 with negative CTP exhibited significant improvement within a week. It is essential to understand that improving CTP-negative patients post-PLF repair surgery does not negate the possibility of perilymph leakage. This is attributed to the potential limitations of the CTP test, which may produce negative results in scenarios of sporadic leakage or minimal perilymph presence in the middle ear during the test. Therefore, due to the test’s constraints, these instances could represent either genuine PLF-negative cases or undiagnosed PLF cases. This insight is vital when analyzing CTP test results within the framework of our research. Most importantly, despite a low positivity rate, it suggests the detectability of perilymph leakage in this cohort. This evidence supports the hypothesis that PLF could be responsible for disequilibrium and related auditory disturbances in these patients.

The immediate relief of vestibular symptoms following PLF repair surgery has not been extensively documented in existing medical literature. However, our observations have been corroborated by neuro-otologists worldwide who specialize in performing this surgery. The underlying pathomechanisms are hypothesized based on detailed anatomical studies. Smith and colleagues have provided a comprehensive description of the intricate structure of the membranous labyrinth in their 2021 study (13). The utricular macula is attached to the bony labyrinth by a perforated membrane, the membrana limitans, giving it some mobility. It is plausible that abnormal endolymph flow due to a window leak may magnify utricular mobility, leading to aberrant vestibulospinal signals that result in unilateral postural instability (14, 15). The fact that repair of the leak ameliorates this symptom aligns with this proposed mechanism and thus should not be surprising. There is a prevalent theory that the vestibular symptoms associated with PLF are due to endolymphatic hydrops (EH) (16), and our current data does not completely deny this theory. However, in a recent Gd-enhanced MRI study showed EH is not a rare finding in the inner ear and is not related to vestibular symptoms. Laine et al. described a 3% incidence of EH in otosclerosis patients and there was no correlation with vestibular symptoms (17). Yoshida et al. reported an incidence of EH in a control group consisting of patients with parotid gland tumors, laryngeal diseases, and sinusitis, who did not exhibit vestibular and cochlear symptoms. EH was identified in 16 out of 42 ears (38%), with significant hydrops observed in 4 out of 42 ears (10%) (18). In addition, the hydrops theory fails to account for the rapid improvement of a patient’s vestibular symptoms after repair of a perilymph leak. The vestibular symptoms from a superior canal dehiscence can be treatable in some cases by “reinforcing” the oval and round windows. However, we do not believe otic capsule dehiscence cases were mistakenly included in the present study. As explained in the methods section, before enrolling patients, we evaluated CT scans to exclude any instances of otic capsule dehiscence. Additionally, VEMPs were tested in 10 of the current cases, revealing hypofunction in the affected ear; none exhibited hyperreactivity.

Two of the 22 cases displayed persistent vestibular and cochlear symptoms and failed to achieve “marked improvement” within the 6-month observation period. Case 13 presented with an episode of vomiting, followed by the onset of vestibular symptoms and auditory impairment. Suspecting an inner ear hemorrhage based on the patient’s MRI findings and ongoing anticoagulant therapy due to a previous myocardial infarction, it was anticipated that the PLF repair surgery would not result in symptom improvements. The link between poor prognosis and sudden sensorineural hearing loss associated with inner ear hemorrhage has been previously documented (19). Case 19’s MRI detected fibrosis in the affected side’s posterior semicircular canal, corroborated by a vHIT revealing impaired functionality. Autoimmune diseases or infarctions within the inner ear can cause inner ear fibrosis (20, 21), but the cause of fibrosis in this case remains unclear. In both 2 cases (13 and 19), vHIT showed a reduction in VOR gain on the affected side, and the compensation process still may need time to heal. The DHI evaluated vestibular symptoms, revealing a statistically significant post-surgical improvement (Figure 1). Three cases sustained a DHI score over 10 within the observation period. Cases 13 and 19, as described above, showed pathologies on the MRI that aligned with their DHI scores of 48 and 42, respectively. Case 22, with a positive CTP test, reported marked improvement within a week following surgery. However, 6 months post-operation, a high DHI score of 68 persisted. The patient was diagnosed with Persistent Postural-Perceptual Dizziness (PPPD) and is presently engaged in cognitive behavioral therapy as part of the treatment protocol (22). The surgical indication for PLF employed in this study were indirectly supported by the positive postoperative outcomes, except for three cases diagnosed with apparent comorbidities, suggesting their overall effectiveness.

The timing of receiving CTP test results is an essential factor when deciding to perform PLF repair surgery. Under the current CTP test architecture covered by the Japanese national health insurance, it typically takes 1 to 3 weeks to receive the results from the central pathology lab. This delay means that decisions about surgery are often made based on other factors, such as the patient’s history, symptoms, and physiological test results. Only 2 cases in this study (cases 11 and 22) had CTP test results available before surgery, as these patients requested to know the results before deciding to undergo surgery. A new point-of-care immunochromatography test to detect CTP is currently under development, which could provide faster and on-site test results. The availability of such a test could lead to more rational surgical indications for PLF repair surgery.

With regard to auditory recovery, cases displaying an improvement of 10 dB or more had a significantly shorter period between onset and surgery compared to those with no response. This aligns with existing literature underscoring the importance of prompt PLF repair surgery (23, 24). In our study, two instances (Case 10 and Case 15) where surgery was conducted more than 3 months post-onset, achieved a postoperative hearing improvement of over 10 dB. In both scenarios, despite a lack of progress with conservative treatment, auditory function improved following surgery. This suggests that auditory improvement is likely attributable to the PLF repair surgery. In situations with a known cause of onset, anticipated severe hearing loss with a poor prognosis, or worsening or fluctuating auditory function, the potential therapeutic benefits of PLF repair surgery should be articulated to the patient, and the appropriateness of surgery should be carefully evaluated.

This study carries several weaknesses. The present study is limited by the absence of a control group, either non-surgical or receiving sham surgery, which introduces the potential for a placebo effect on subjective vestibular symptoms. Regardless, our findings demonstrated that even among the 11 cases presenting chronic phase vestibular symptoms (cases 10 through 15, and 18 through 22, each persisting beyond 3 months), 9 cases (or 82%) exhibited marked improvement within one-week post-surgery. This supports the inference that the observed gains are likely attributable to the surgical intervention.

## Conclusion

From our current study, we observed rapid and marked improvements in vestibular symptoms and significant reductions in DHI scores after PLF repair surgery. These results highlight the high efficacy of this procedure in treating PLF, provided that patients are appropriately selected based on clinical findings. Additionally, when the surgery was performed early, we noticed improvements in hearing. Out of our sample, the CTP test was positive in four cases and intermediate in another four. Although the positivity rate was low, it indicates that perilymph leakage is detectable in this population. This evidence supports the hypothesis that PLF could be responsible for disequilibrium and related auditory disturbances in these patients.

The effectiveness of PLF repair surgery for PLF can be influenced by factors such as the size of the fistula, the extent of perilymph leakage, the number of days to surgery, and the severity of inner ear damage at the onset. Conducting a prospective study using our developed surgical indication will further elucidate PLF’s symptoms and treatment efficacy.

## Data availability statement

The raw data supporting the conclusions of this article will be made available by the authors, without undue reservation.

## Ethics statement

The studies involving humans were approved by Institutional Review Board of Saitama Medical University Hospital. The studies were conducted in accordance with the local legislation and institutional requirements. Written informed consent for participation in this study was provided by the participants’ legal guardians/next of kin.

## Author contributions

HM: Writing – original draft. JH: Writing – review & editing. TI: Conceptualization, Writing – original draft.
