# Supplementary material for: Assessing the efficacy of perilymphatic fistula repair surgery in alleviating vestibular symptoms and associated auditory impairments
Source: Front Neurol. 2023 Oct 12;14:1269298. doi: 10.3389/fneur.2023.1269298 (PMC10600483; doi:10.3389/fneur.2023.1269298)
Supplement: Supplementary file 1 [file Table_1.docx]

Supplementary Table

|  | Overall (22 cases) | Group 1 | Group 2 |
| --- | --- | --- | --- |
| Surgery in Acute Phase (within 2 weeks) | 5 | 5 | None |
| Chronic Vestibular Symptoms (>90 days) | 10 | 5 | 5 |
| Initial Vestibular Symptoms |  |  |  |
| Rotatory vertigo to Disequilibrium | 8 | 5 | 3 |
| Disequilibrium at onset | 14 | 10 | 4 |
| Nystagmus Resolution (numbers indicating cases where nystagmus was present at each visit.) |  |  |  |
| Pre-surgery Nystagmus | 16 | 12 | 4 |
| Post-surgery 1 week | 8 | 7 | 1 |
| Post-surgery 1 month | 2 | 2 | 0 |
| Post-surgery 3 months | 2 | 2 | 0 |
| Post-surgery after 6 months | 2 | 0 | 2 |
| *Cases 13, 19 continued to exhibit nystagmus. |  |  |  |
|  |  |  |  |
| Type of Hearing Loss | Overall (20 cases) | Group 1 | Group 2 |
| Sudden onset | 9 | 4 | 5 |
| Fluctuating | 3 | 3 | 0 |
| Progressive | 8 | 6 | 2 |
